# Measurement of Hydraulic Fracture Aperture by Electromagnetic Induction

**DOI:** 10.3390/s24206660

**Published:** 2024-10-16

**Authors:** Mohsen Talebkeikhah, Alireza Moradi, Brice Lecampion

**Affiliations:** 1 Institute of Civil Engineering, Gaznat Chair on Geo-Energy, École Polytechnique Fédérale de Lausanne (EPFL), 1015 Lausanne, Switzerland; m.talebkeikhah@gmail.com; 2 Faculty of Engineering, Tarbiat Modares University (TMU), Tehran 14115-111, Iran; amoradi@blueheartenergy.com

**Keywords:** fracture mechanics, hydraulic fracturing, aperture measurement, porous sandstone, eddy current probe, eddy current, fracture opening, fracture width

## Abstract

We present a new method for accurately measuring the aperture of a fluid-driven fracture. This method uses an eddy current probe located within a completion tool specifically designed to obtain the fracture aperture in the wellbore at the location where the fluid is injected into the fracture. The probe induces an eddy current in a target object, producing a magnetic field that affects the overall magnetic field. It does not have any limitations with respect to fluid pressure and temperature within a large range, making it unlike other methods. We demonstrate the accuracy and performance of the sensor under laboratory conditions. A hydraulic fracture experiment in a porous sandstone is conducted and discussed. The obtained measurement of the evolution of the fracture inlet aperture by the eddy current probe during the multiple injection cycles performed provided robust information. The residual fracture aperture (after the test) measured by the probe is in line with estimations from image processing of X-ray CT scan images as well as a thin-section analysis of sub-parts of the fractured specimen. The robustness and accuracy of this electromagnetic induction probe demonstrated herein under laboratory conditions indicate an interesting potential for field deployment.

## 1. Introduction

Hydraulic fracturing is widely used to enhance production from oil and gas wells. It consists of creating fractures within a reservoir, which are then propped open by the injection of sand to improve the overall hydraulic transmissivity of the reservoir in the area surrounding the well. Fractures primarily govern fluid flow within tight rock formations, essential in various applications like oil and gas extraction, geothermal energy production, ${\mathrm{CO}}_{2}$ sequestration, and underground nuclear waste storage. A precise understanding of how fractures propagate under fluid injection is critical for the efficiency and safety of these geo-energy applications. Understanding fracture aperture and its variability is essential for validating hydraulic fracture models and comprehending the extent of fracturing and the characteristics of the proppant used to keep the fracture open.

Records of the injection fluid pressure are the simplest measurements available to understand fracture propagation. It, however, provides only partial information and is often insufficient to properly assess the growth of hydraulic fractures. Active and passive seismic monitoring are often used to estimate the geometrical extent of the fracture [1,2,3,4] with more or less accuracy depending on the measurement location. The measurement of the induced elastic strain or rotation associated with hydraulic fracture propagation at distant locations from the created fracture provides a robust estimation of fracture volume and orientation. However, it lacks sensitivity to fracture shapes if measurements are not located in the near field [5,6]. Direct and accurate measurements of fracture aperture are particularly challenging in non-transparent materials, like rocks, but would provide important additional information to better monitor fracture propagation.

Some measurement methods can measure the fracture shape and aperture profile. For instance, Bunger [7] developed a photometry method for measuring fracture aperture in transparent materials. Groenenboom et al. [8,9,10] observed the dispersion of compressional waves across hydraulic fractures, which is indicative of fracture width and suitable for thin fluid-filled layers but necessitates both compressional and shear wave measurements. Furthermore, geophysical imaging techniques, such as seismic reflection, electrical methods, and ground-penetrating radars, have been utilized to detect subsurface features [11].

Distributed strain measurements from fiber optics placed in wellbores have recently been deployed in hydraulic fracturing operations. Chen et al. [12] developed a fiber optic sensing scheme to image 3D strain fields in concrete during hydraulic fracturing. Leggett et al. [13] and Yang et al. [14] employed hydraulic fracture experiments with embedded optical strain sensors and fiber Bragg grating strain sensors, respectively. Qasim et al. [15] conducted a field data analysis using distributed fiber optic technology in various international basins. Additionally, precise point-wise measurement methods exist but can only measure the aperture at specific points, such as injection points. Kakurina et al. [16] used a specific fiber-optic probe (the SIMFIP probe) to measure 3D displacement at a given location in boreholes. Additionally, De Pater et al. [17] used a Linear Voltage Differential Transformer (LVDT) to measure fracture opening during propagation and closure in the laboratory. A similar technique was used by Warpinski [18], who measured fracture width during hydraulic fracture propagation using LVDTs in boreholes at a U.S. Nevada test site. Furthermore, Overbey et al. [19], Palmer and Sparks [20], Darilek [21] utilized borehole video cameras or television systems to “estimate” fracture openings. Smith et al. [22] conducted measurements using a downhole closed-circuit television camera during hydraulic fracturing propagation stimulation in an oil-bearing sandstone formation. Moreover, Medlin and Masse [23] utilized a capacitance bridge to continuously measure fracture aperture in a cubic sandstone specimen in the laboratory.

In this study, we propose a robust method for directly measuring the aperture at the fracture inlet. We present its mechanism and calibration procedure under various conditions. Subsequently, we conduct a laboratory-scale hydraulic fracture experiment on a cubic sandstone block. Throughout these experiments, we monitor both pressure and aperture at the inlet of the fracture within the rock sample. The obtained results will be validated by comparing the probe measurement with the sample’s 3D X-ray computer tomography (CT) scan and thin-section analysis, demonstrating the method accuracy and reliability.

## 2. Problem Description

The main goal of this research is to conduct a laboratory-scale hydraulic fracturing experiment and evaluate the practicality of a new method for measuring fracture aperture at the fracture inlet. Our approach involves placing a probe directly at the point where the fluid is injected to measure the fracture aperture. This injection point is positioned at the center of a cubic sandstone block in the middle of a drilled well within the specimen. However, there are several challenges in this process. The drilled well has a diameter of approximately 20 mm, requiring the completion tool, which contains the probe as well as the fluid injection line, to be compact yet accessible at a depth of 125 mm.

Additionally, the measurement process must be sufficiently fast to capture the real-time evolution of fracture aperture during an experiment. It is essential to maintain a high level of accuracy and acquisition frequency. The probe must precisely measure fracture apertures on the order of micrometers (μm), with an accuracy below 1 μm, and the measurement frequency must be sufficient to record all events. Moreover, the probe must withstand high pressures, as injection fluid pressures can exceed 60 MPa, be sealed to the injection fluid, and resist any chemical reactions from the components. Lastly, our design must allow for the probe’s reusability by making it easy to remove from the completion tool after the experiment.

## 3. Gap Measurement via Electromagnetic Induction

### 3.1. Probe Description

An electromagnetic inductive probe called an Eddy Current (EC) probe is a type of contact or non-contact probe that utilizes electromagnetic induction to measure a target object’s position, displacement, or distance. This probe possesses several advantages, such as high sensitivity, strong anti-interference ability, and resistance to oil, water, and other media, ensuring reliable operation over extended periods. The probe’s non-contact measurement and rapid response make it suitable for long-term, real-time monitoring. It is based on the principle that when a magnetic field is introduced into a conductive material, it will induce eddy currents (according to Faraday’s law of electromagnetic induction) that generate their magnetic field, which, according to Lenz’s law, opposes the original magnetic field. As a result, there is a change in the overall magnetic field and, accordingly, the impedance value of the coil in the probe. The magnitude of this change is directly proportional to the distance between the probe and the target object [24]. The eddy current probe has numerous applications in various industries, such as industrial automation, aerospace, and automotive. For instance, it can be used to inspect metal parts, measure the thickness of metal plates, and track the position of moving machinery.

**Figure 1:**
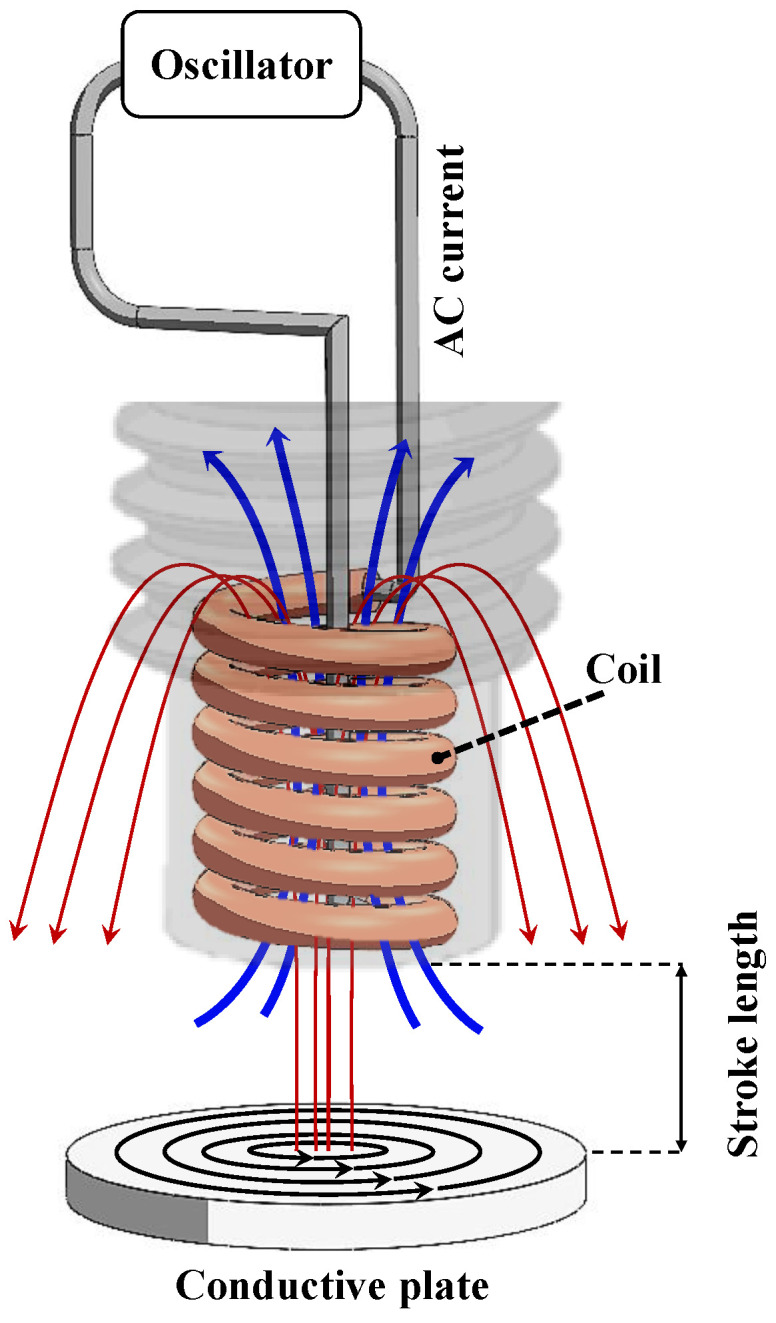
Schematic representation of the operational mechanism of an eddy current (EC) probe.

The mechanism employed by the eddy current probe to measure distance, as illustrated in Figure 1, involves producing an alternating current (AC) in a coil of wire inside the probe through an oscillator. This current generates a magnetic field (blue lines), which induces eddy currents (black lines) in the conductive plate. The eddy currents then produce their magnetic field (red lines), which superimposes the original magnetic field produced by the coil [25]. The resulting interaction changes the coil’s impedance, which can be measured and utilized to determine the distance between the probe and the plate. The probe’s electronics convert this measurement into an analog or digital signal that a computer or other control system can read. In this study, a Miran eddy current probe, provided by Miran Technology Co., Ltd. (Shenzhen, China) (http://www.miransensor.com/ accessed on 1 September 2022), was utilized. Table 1 summarizes the main properties of this EC probe.

### 3.2. Calibration

#### 3.2.1. Calibration Setup

**Figure 2:**
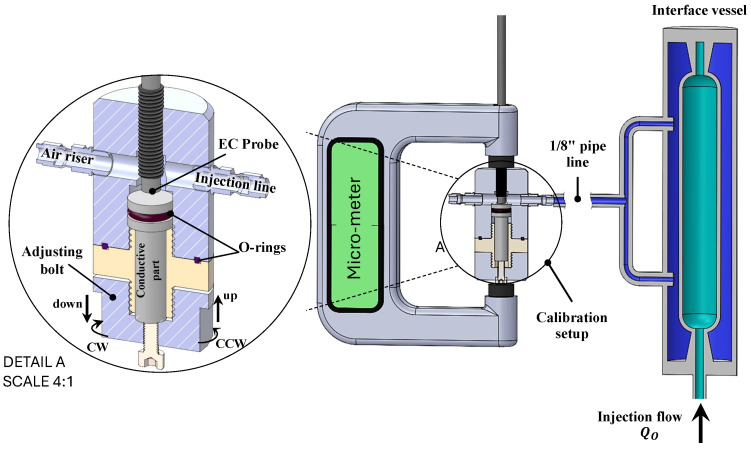
Schematic of the setup designed for calibrating the EC probe with the condition of the hydraulic fracturing experiment.

Large fluid pressures may affect the behavior of the EC probe during hydraulic fracturing experiments. Additionally, the choice of the conductive plate material, whether steel, iron, or aluminum, also impacts the EC probe readings. In all cases, it is essential to calibrate the EC probe’s linear output by measuring the slope of the linear function between the EC probe value and the corresponding physical distance. To do so, a calibration device has been designed and built as shown in Figure 2. This calibration device positions the EC probe in front of an aluminum conductive plate that simulates the conditions of the primary experiment. The conductive plate can be moved vertically by rotating the bottom part of the device along its thread, creating a controlled movement in front of the EC probe tip that can be compared to the EC probe reading. The system is connected to an injection line, an interface vessel containing injection fluid (glycerol), and an air riser line that removes air during fluid pressurization. A specialized micrometer is attached to the calibration device, which can accurately measure the exact distance variation in the system. This length difference corresponds to the vertical movement caused by the bottom rotation and conductive parts around the thread. The volume between the EC probe and the conductive plate is pressurized by fluid injection during the calibration test. The injection fluid is sourced through a 1/8″ (in) line tube connected to an interface vessel. This interface vessel is then connected to a high-pressure pump that injects water at a constant rate/pressure. The interface vessel conveys the water pressure from the pump to the injection fluid, which can be glycerol, glucose, silicon oil, or any other suitable fluid. At various constant pressures, the manual rotation of the calibration device’s bottom part creates a controlled displacement between the EC probe and the conductive plate. The EC probe output value is recorded alongside the corresponding displacement value measured by the micrometer, providing a correlation between the EC probe readings and the actual displacement for each pressure level.

#### 3.2.2. Calibration Results

The calibration device filled with glycerol is intended to be used during the hydraulic fracturing test. The pressure was kept constant during each calibration phase, during which the bottom part of the calibration device was manually rotated. The corresponding values from the micrometer and EC probe readings are recorded while rotating the bottom part until the EC probe reading changes from 0 to 1. This procedure is repeated several times for different fluid pressure values from 0.1 to 20 MPa.

**Figure 3:**
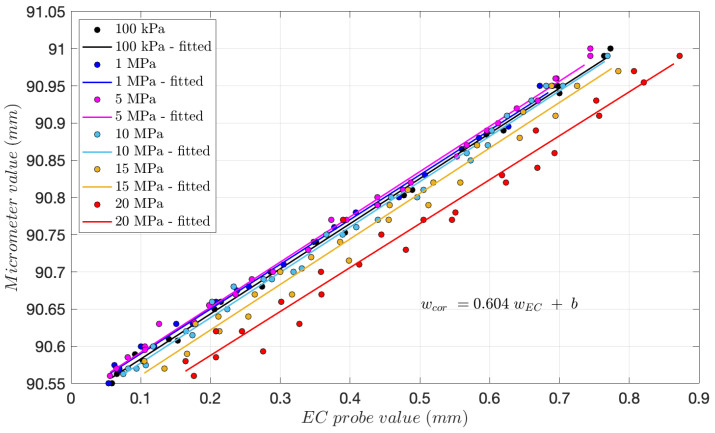
EC probe readings and corresponding micrometer results obtained in the calibration device at various fluid pressures of 0.1, 1, 5, 10, 15, and 20 MPa.

The results of this calibration are reported in Figure 3. Each set of constant pressure data was fitted with a line, revealing that all lines share the same constant slope. The difference in the intercept value (*b*) between the lines for different pressures can be attributed to the calibration device’s deformation, especially at higher pressure levels.

This is inconsequential, as the difference between the current and the initial EC probe reading (prior to the start of fluid injection and fracturing) is taken to determine the aperture of a hydraulic fracture at the wellbore during the hydraulic fracturing experiment. Therefore, the required calibration is solely given by the slope value of the EC probe–micrometer value m=0.604. Afterward, the EC probe is placed within the completion tool at the injection point inside the rock, as detailed in the next section. After conducting the experiment, the obtained EC probe values are multiplied by the slope value of the line to obtain the actual fracture aperture values.

## 4. Hydraulic Fracture Experimental Setup

### 4.1. Materials

A cubic block of Molasse sandstone was selected to conduct a hydraulic fracturing experiment on a laboratory scale. The sandstone was sourced from the Molasse de Villarlod quarry in Switzerland and had a nominal length of 250 mm. This sandstone sample is a porous and permeable rock, which makes it an ideal choice for the experiment. By selecting a porous and permeable sample, the experiment aimed to simulate the opening and closure of a fracture, as the fluid inside the fracture would diffuse into the surrounding porous media.

### 4.2. Experimental Setup Description

**Figure 4:**
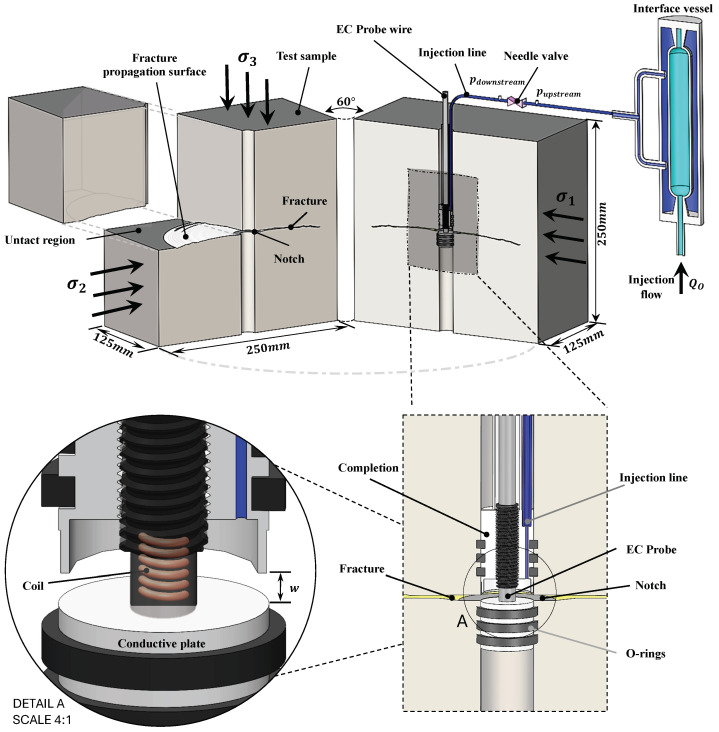
Schematic of the experimental setup for the propagation of hydraulic fracture under confinement, with details of the completion and the EC probe to measure fracture aperture at the fracture inlet.

The experimental setup consists of a polyaxial frame designed to investigate hydraulic fracture propagation under a true-triaxial state of stress. A schematic of the setup is given in Figure 4. Triaxial confining stresses are applied via three independent symmetric pairs of flat-jack ($\sigma_{3}$) and pistons ($\sigma_{1},\;\sigma_{2}$). The fracturing fluid is injected through a high-pressure line $1/8^{\prime \prime }$ in diameter, cemented with epoxy in a centrally drilled hole within the block. An axisymmetric notch is created at the middle of the well to facilitate fracture initiation and enhance fracture planarity. A planar hydraulic fracture is then propagated by injecting high-pressure viscous fluid from the notch to the sample’s boundary. Throughout the experiment, injection fluid rates and pressures are continuously measured. We refer to [3,26,27,28,29] for a detailed description of the experimental setup. The main addition discussed here relates to the addition of an EC probe inside the central wellbore.

### 4.3. Installation of the EC Probe in the Wellbore

The EC probe introduced in Section 3.1 is positioned in the central wellbore right at the position of the notch, precisely where the fluid is injected into the rock. The EC probe, therefore, shall provide a precise measurement of the fracture aperture at the injection point. A specialized completion tool aligns the probe’s tip with the notch, simultaneously accommodating the injection fluid line. This specifically designed completion tool is depicted in Figure 4. It consists of an upper and a lower part. The upper part, made from stainless steel, centrally holds the probe while the injection line is positioned eccentrically. The lower part, made of aluminum, is the EC probe’s conductive plate and target object. As the fracture initiates and its aperture expands, the distance between the EC probe and the conductive plate increases. Consequently, the induced eddy currents in the conductive plate decrease and the probe measures this distance, which corresponds to the aperture of fracture, in real time throughout the experiment.

## 5. Results and Discussion

### 5.1. Overview of the Experiment

As an illustrative example, we report a hydraulic fracture experiment performed in a porous sandstone rock from the Molasse de Villarlod quarry in Switzerland. This experiment uses glycerol as a fracturing fluid with a viscosity of μ=0.57 Pa·s measured by a rotational cone and a plate viscometer. Fluorescence additives were dissolved into the glycerol fluid to visualize the fracture path under UV light, facilitating easy observation of the fracture once the sample was cut after the experiment.

**Figure 5:**
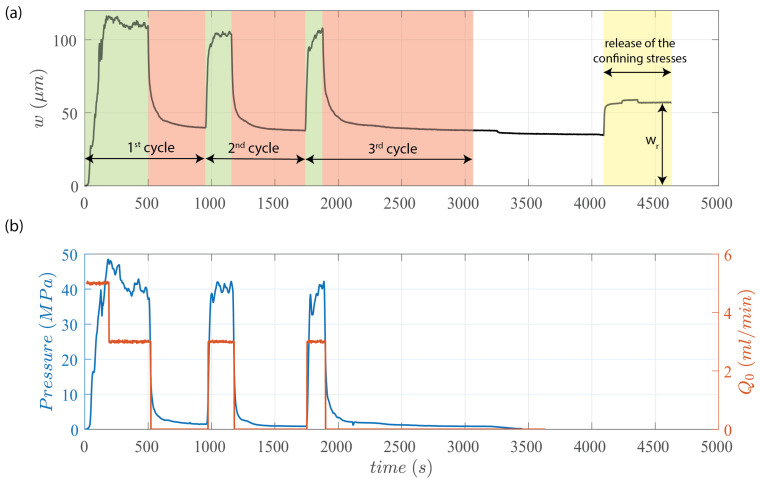
Hydraulic fracturing experiment in a porous sandstone. (**a**) Time evolution of the fracture aperture at the wellbore measured via the EC probe (in black), (**b**) time evolution of the fluid pressure in the well (in blue) and the injection rate (in red). Three injection cycles were performed. After a hold period following the last cycle, the applied confining stresses were released.

After the preparation of the sandstone block for the experiment and the placement of the EC probe inside the completion tool, we initially subject the block to lateral confining pressures of $\sigma_{1}\;=\;\sigma_{2}\;=\;15\;$ MPa and a vertical confining pressure of $\sigma_{3}\;=\;5$ MPa. This ensures that the created fracture will propagate horizontally in a radial manner from the notch. Additionally, before starting the injection of the glycerol, we saturate all fluid lines with it to avoid air bubbles. The records of the fluid injection pressure and rates, as well as of the measurement of the wellbore fracture aperture *w*, are reported in Figure 5. In Figure 5a, the fracture aperture over time at the injection point is plotted in black, while Figure 5b depicts the fluid pressure in blue and the injection rate in red over time. It is important to note that the confining stresses were applied early on (at t = 0 s), and the reading of the EC probe is “zeroed” just after.

As reported in Figure 5, glycerol was injected into the central wellbore at a rate of 5 mL/min starting at time t = 0 s. However, due to the large pressurization rate, the fluid injection rate was reduced to 3 mL/min to avoid reaching the maximum pressure that the injection pump could handle. The fluid pressure largely exceeded the minimum applied confinement, and subsequently dropped while the fluid was still injected, clearly indicating the propagation of a fracture [30].

The fracturing continued until 462 s, when a needle valve in the injection was closed, suddenly stopping the fluid injection. As a result, fracture propagation stopped, and the hydraulic fracture began to recess and close. The sandstone is porous and permeable, allowing the fluid within the fracture to diffuse into the surrounding porous medium. This results in a decrease in fluid pressure in the injection line and fracture, thus decreasing the wellbore fracture aperture.

Subsequently, after some time, the fluid injection was re-started at 937 s with a rate of 3 mL/min, then stopped. This was repeated once more, resulting in three complete cycles of fluid injection, fracture creation and propagation, closure of the needle valve, cessation of fluid injection, and fracture closure. In Figure 5a, the highlighted green sections correspond to the fluid injection and fracture propagation periods. In contrast, the highlighted red sections indicate the periods without any injection where the fracture recesses and closes. Finally, at 2135 s, the needle valve is opened, but no further injection is performed. The experiment concludes. From 2944 to 3267 s, the applied confining pressures of 15 MPa and 5 MPa are released (time period highlighted in yellow in Figure 5a). This results in an elastic rebound of the measured fracture aperture in the wellbore, as measured by the EC probe (see Figure 5a).

**Figure 6:**
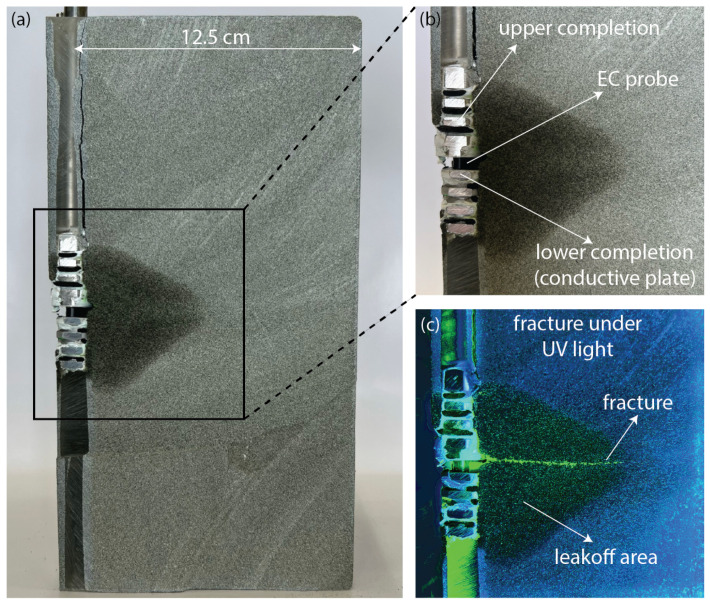
(**a**) Sandstone sample sliced into four parts after the experiment, displaying the wellbore, completion tool, and the EC probe. (**b**) A closer view of the area surrounding the fracture inlet, where the EC probe is located. (**c**) The same section is exposed to UV light, revealing the fracture path and the leakoff area.

Once the experiment is completed, the sandstone sample is cut in order to visually inspect the created fracture. The sample is sliced into two vertical cuts parallel to the wellbore axis, resulting in four parts. Figure 6 displays one of these parts, including the wellbore and the completion tool, in Figure 6b, a closer view of the area surrounding the wellbore inlet is provided, where the EC probe is located. The upper and lower completion and the EC probe are visible. In Figure 6c, the same section is exposed to UV light, revealing the fracture path and the leakoff area where the fracturing fluid has diffused inside the porous rock.

### 5.2. Discussion

**Figure 7:**
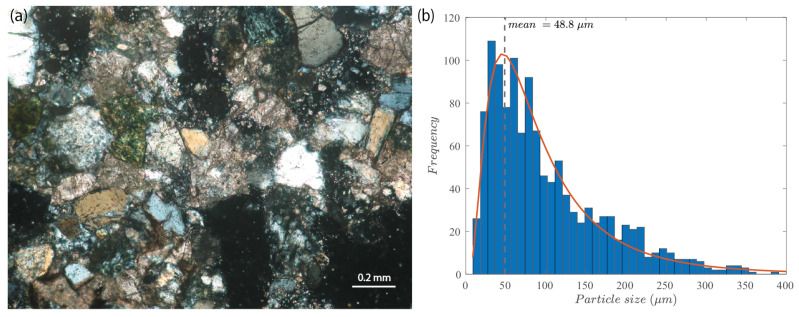
(**a**) Thin-section image depicting the microstructure of the Molasse sandstone. (**b**) The 2D particle size distribution of the sandstone obtained from image processing of the thin section.

It is important to note that even after completely depressurizing the sample, the measured fracture aperture at the wellbore did not return to zero. This means that the initiation, propagation, and closure of the hydraulic fracture leaves a residual aperture in the rock. Such a residual fracture aperture has been reported in numerous experiments [8,9,18,23]. In the experiment reported herein, as highlighted in the yellow section of Figure 5a, the residual fracture aperture $w_{r}$ after all the stresses applied to the sample were released was about 57 μm according to the EC probe reading. This residual aperture could be related to the characteristic grain size of the rock. During the fracturing process, grains induce a deviation from planarity, and a slight misalignment of the fracture surfaces necessarily occurs upon closure. A thin section of the Molasse sandstone can be seen in Figure 7a. An image processing code has been developed to obtain the particle’s size distribution in 2D (see panel b in Figure 7). It follows a classical log-normal distribution, with a mean particle size of 48.8 μm, on par with the measured residual aperture $w_{r}$ from the EC probe (57 μm).

**Figure 8:**
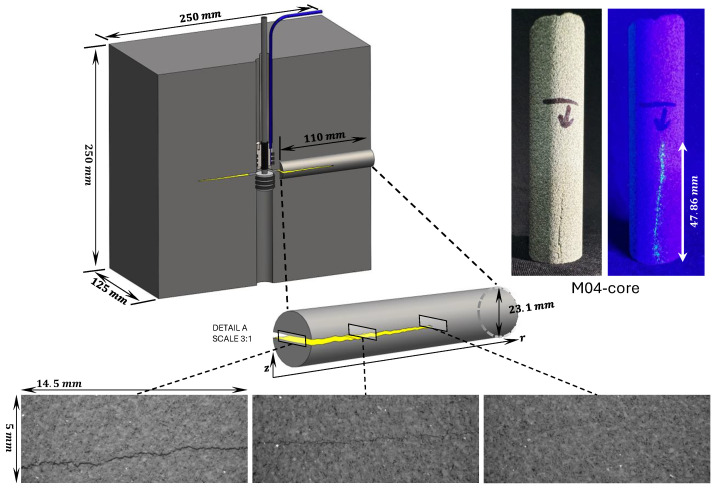
Schematic diagram of the rock sample with core extraction and location of the corresponding CT scan images taken near the fracture inlet, in the middle part of the fracture, and near the fracture front. The corresponding core sample images are in the right section.

**Figure 9:**
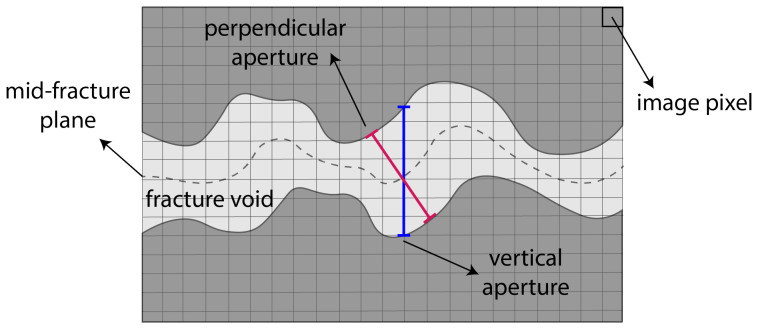
Comparison of the aperture of the normal fracture to the mid-plane fracture with the vertical aperture of the fracture.

We also performed an additional inspection of the fractured specimen after the test. A core was drilled in the sandstone specimen within a zone containing the created fracture. This core spans a radial zone from the central notch to the boundary of the sample (see Figure 8 for a schematic). An X-ray microcomputer tomography (CT) scan of this core was performed at the PIXE facility at EPFL. This CT scan process involved capturing 2D images of the core in a plane perpendicular to the fracture surface. To do so, CT scans were taken every 10 microns (the adjusted resolution of the CT scan instrument) in the mentioned direction, from the notch to the boundary of the sample. Figure 8 depicts the rock sample along with the core extracted schematically. Furthermore, three example images are reported at the locations of the fracture inlet (near the notch), fracture middle part, and fracture tip. In all three CT scan images depicted in Figure 8, a noticeable dark and rough area can be observed. This area, in each CT scan image, corresponds to the fracture voids, thus allowing us to estimate the fracture aperture. This aperture is the residual fracture aperture because it remains in the specimen after the fracturing experiment with all loadings removed. Based on the information obtained from the CT scan instrument, it has been determined that the size of each pixel in the CT scan image is 10 microns. This detail can be used to estimate the size of the fracture aperture by counting the number of pixels that correspond to the dark area. It is important to note that due to the rough surface of the fracture, the aperture normal to the mid-fracture plane is used to measure the average fracture aperture in each CT scan image, rather than the vertical extent of the fracture void (referred to as the “vertical” aperture here). The difference between the normal aperture and vertical aperture is depicted in Figure 9.

Manually estimating the average perpendicular aperture for each CT scan image is impractical due to the large number of images—over 1000 per centimeter. Automatic image processing was, therefore, developed to do so. Its algorithm uses image processing techniques to calculate the average perpendicular aperture for all CT scan images. The process begins with reading TIFF images provided by the CT scan instrument, whose pixel values are then normalized between 0 and 1. A median filter is then applied to eliminate noise, and a contrast-limited adaptive histogram equalization is utilized to enhance the contrast of the grayscale image. Following this, a threshold is applied to remove all connected components that have fewer pixels than a specific number from the binary image (area opening operation). Finally, the Canny edge detection method is applied to identify the darker area edges and find the row and column indices of the non-zero elements (edges). These CT scan image datasets are available for download at M04 core CT scan images (https://doi.org/10.5281/zenodo.13357511 accessed on 1 September 2024), and the code for processing these CT scan images is available for access at FractureSurf (https://github.com/GeoEnergyLab-EPFL/FractureSurf/ accessed on 1 September 2024).

**Figure 10:**
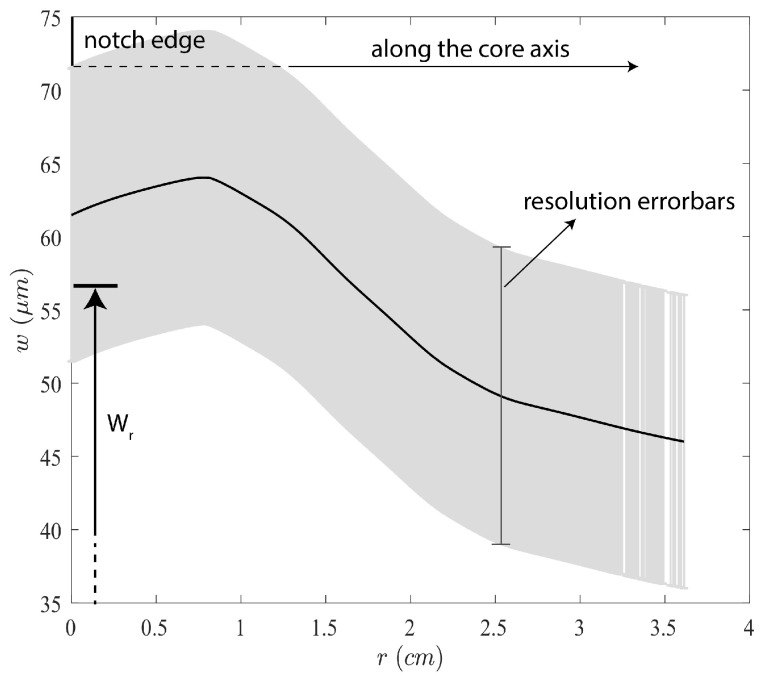
Profile of the residual aperture of the fracture along the length of the core sample.

In this way, an average value for the perpendicular aperture can be estimated for each CT scan image. By plotting the average aperture value of each CT scan image as a function of its position from the central wellbore, it is possible to obtain a comprehensive profile of the average aperture of the fracture along the length of the core. This profile extends from the notch to the fracture front and is shown in Figure 10. The gray area in this figure represents the error bars associated with the adjusted resolution of the CT scan. The residual aperture at the notch measured by the EC probe at the end of the experiment (in Figure 5a) of 57 microns falls within the range of 53–73 microns of the CT scan estimations (Figure 10). Both estimations correspond to similar conditions, without fluid and confinement pressures, which indicates that the EC probe and CT scans provide similar measurements of the fracture aperture.

### 5.3. Additional Requirements for Field Applications

The research conducted in this study demonstrated the very good performance of the EC (eddy current) probe, which was able to produce accurate results. The characteristics of the sensor, which are presented in Table 1, include high pressure and high temperature tolerance, as well as immunity to all types of fluids. Therefore, it could be implemented at field scale in harsh downhole environments at a depth typical of geo-energy applications (1 to 5 km). Specific arrangements must be made to deploy such a measurement at field scale. For instance, it will be necessary to clamp the target conductive plate (lower completion) and the EC probe holder (upper completion) to the walls of the well to place them on both sides of the fracture while allowing vertical relative movement. This would enable measuring the gap changes between the probe and the target plate, which corresponds to the aperture of the fracture. In addition, a stronger EC probe with a larger stroke length should be selected in order to measure a larger fracture aperture. If running a wire connection between the bottom and top of the well is not feasible, a downhole electronic system for the probe will have to be developed. This system should not only store the measured signals but also provide the energy required for the probe’s operation.

The erosion of the probe and conductive plates by, for example, proppant used in hydraulic fracturing operations could be mitigated by coating the probe and conductive plates with more durable materials, such as diamond-like carbon. Alternatively, a layer of compacted protective material can be placed around them. Additionally, a stiffer metal could be used for the target plate to further enhance durability. In any case, proper probe calibration according to the material of the target conductive plate and the type of injected fluid, as conducted in this study, will always be required.

## 6. Conclusions

In this study, we successfully integrated an eddy current (EC) probe into a specialized completion tool positioned in a wellbore to accurately measure the fracture aperture during high-pressure fluid injection. Our findings demonstrate the reliability and applicability of this method to monitor the evolution of fracture aperture throughout hydraulic fracture initiation, propagation, and closure (once the injection has stopped). The residual fracture width measurements obtained from the EC probe at the conclusion of the fluid injection, after the stresses were removed, align with those derived from X-ray CT scan images and thin-section analysis. We have observed that the EC probe performs exceptionally well in laboratory settings. It is, therefore, a promising technique with potential applications in larger-scale field conditions. However, achieving this will require the development of a specifically designed EC probe and a hardware mounting tool to enable placement and retrieval in deep wellbores.

## Figures and Tables

**Table 1:** Main specifications of the EC probe used in this study.

Property	Value	Unit	Property	Value	Unit
Stroke length	1	mm	Probe diameter	6	mm
Probe length	5	mm	Temperature range	−30∼150	C
Resolution	0.05	μm	Output signal range	0∼5	V

## Data Availability

The dataset, which includes detailed measurements from a radial hydraulic fracturing experiment conducted on a cubic M04 sample, is available for download at M04 Radial Hydraulic Fracturing (https://doi.org/10.5281/zenodo.13402755 accessed on 1 September 2024). The CT scan images of the present study (M04 core sample) are available for download at M04 core CT scan images https://doi.org/10.5281/zenodo.13357511 accessed on 1 September 2024. The code for processing low-rate data, including opening and pressure measurements, is available for access at FracLowRate https://github.com/GeoEnergyLab-EPFL/FracLowRate accessed on 1 September 2024. The code for processing the CT scan images is available for access at FractureSurf https://github.com/GeoEnergyLab-EPFL/FractureSurf/ accessed on 1 September 2024.
